# The clinical implications of thrombocytopenia in adults with severe falciparum malaria: a retrospective analysis

**DOI:** 10.1186/s12916-015-0324-5

**Published:** 2015-04-24

**Authors:** Josh Hanson, Nguyen Hoan Phu, Mahtab Uddin Hasan, Prakaykaew Charunwatthana, Katherine Plewes, Richard J Maude, Panote Prapansilp, Hugh WF Kingston, Saroj K Mishra, Sanjib Mohanty, Ric N Price, M Abul Faiz, Arjen M Dondorp, Nicholas J White, Tran Tinh Hien, Nicholas PJ Day

**Affiliations:** Mahidol Oxford Tropical Medicine Research Unit, Faculty of Tropical Medicine, Mahidol University, Bangkok, Thailand; Global and Tropical Health Division, Menzies School of Health Research and Charles Darwin University, Darwin, NT Australia; Oxford University Clinical Research Unit, Hospital for Tropical Diseases, Ho Chi Minh City, Vietnam; Chittagong Medical College, Chittagong, Bangladesh; Centre for Tropical Medicine and Global Health, Nuffield Department of Medicine, University of Oxford, Oxford, UK; Department of Laboratory Medicine, Faculty of Medicine, Chulalongkorn University, Chulalongkorn, Thailand; Dev Care Foundation, Dhaka, Bangladesh

**Keywords:** Bleeding, Patient triage, Severe falciparum malaria, Thrombocytopenia

## Abstract

**Background:**

Thrombocytopenia is a common finding in adults with severe falciparum malaria, but its clinical and prognostic utility is incompletely defined.

**Methods:**

Clinical and laboratory data from 647 adults with severe falciparum malaria were analysed retrospectively to determine the relationship between a patient’s platelet count on admission to hospital and their subsequent clinical course.

**Results:**

On admission, 614 patients (94.9%) were thrombocytopenic (platelet count <150 × 10^9^/L) and 328 (50.7%) had a platelet count <50 × 10^9^/L. The admission platelet count was inversely correlated with parasite biomass (estimated from plasma *Pf*HRP2 concentrations, r_s_ = −0.28, *P* = 0.003), the degree of microvascular sequestration (measured with orthogonal polarizing spectral imaging, r_s_ = −0.31, *P* = 0.001) and disease severity (the number of World Health Organization severity criteria satisfied by the patient, r_s_ = −0.21, *P* <0.001). Platelet counts were lower on admission in the patients who died (median: 30 (interquartile range 22 to 52) × 10^9^/L versus 50 (34 to 78) × 10^9^/L in survivors; *P* <0.001), but did not predict outcome independently from other established laboratory and clinical prognostic indices. The 39 patients (6%) with profound thrombocytopenia (platelet count <20 × 10^9^/L) were more likely to die (odds ratio: 5.00, 95% confidence interval: 2.56 to 9.75) than patients with higher platelet counts, but these high-risk patients could be identified more rapidly with simple bedside clinical assessment. The admission platelet count did not reliably identify the 50 patients (7.7%) with major bleeding during the study.

**Conclusions:**

Thrombocytopenia is a marker of disease severity in adults with falciparum malaria, but has limited utility in prognostication, triage and management.

## Background

Thrombocytopenia is an early and consistent feature of malaria [1-4], but its pathogenesis remains incompletely understood. In falciparum malaria there is increased platelet consumption as evidenced by shortened survival of radiolabelled platelets [5] and the finding of plentiful megakaryocytes in patients’ bone marrow [6] and appropriately elevated plasma thrombopoietin levels [7]. Both systemic microvascular sequestration [8] and endothelial activation [9,10] may play a pathophysiological role, a hypothesis supported by the observation that the radiolabelled platelets of patients with falciparum malaria are diffusely sequestered, rather than pooling in the liver or spleen [5]. Yet while thrombocytopenia is a ubiquitous laboratory finding, it had been thought to have limited clinical significance, as major bleeding is relatively uncommon in the disease [11].

Recently it has been suggested that thrombocytopenia may have important implications for patient triage. Population studies have shown an association between thrombocytopenia and outcome [12] and a recent study from India proposed that thrombocytopenia should be added to the World Health Organization (WHO) criteria for the definition of severe malaria [13]. To clarify this issue, data from adults with severe falciparum malaria prospectively enrolled in clinical studies of severe malaria were analysed to determine if the platelet count could assist in clinical decision-making and whether the degree of thrombocytopenia has predictive utility independent of current clinical and laboratory prognostic indices. Measures of microvascular sequestration and endothelial activation and their relationship with the platelet count were examined to further explore the pathogenesis of the thrombocytopaenia seen in the disease.

## Methods

Two datasets were combined for this analysis. The first (n = 560) was compiled prospectively at the Centre for Tropical Diseases in Ho Chi Minh City, Vietnam, between 1991 and 1996 in a study that compared the efficacy of artemether and quinine in adults with severe falciparum malaria [14]. The second comprised data collected prospectively in studies assessing a range of adjunctive therapies in severe falciparum malaria at Chittagong Medical College Hospital, Bangladesh, and Ispat General Hospital, Rourkela, India, between 2004 and 2011 [15-18].

Malaria transmission was low and seasonal at all sites. Falciparum malaria was diagnosed if a blood film showed asexual forms of *Plasmodium falciparum*. When expert microscopy was not available immediately, patients were enrolled if an immunochromatographic rapid diagnostic test (Paracheck Pf, Orchid Biomedical Systems, Goa, India) was positive; *P. falciparum* infection was confirmed later by microscopy of a simultaneously collected blood slide.

All patients satisfied a strict definition of severe falciparum malaria that used modified WHO criteria [19], including cerebral malaria (Glasgow Coma Scale (GCS) <11); severe anaemia (haematocrit <20% with a parasite count > l00,000/mm^3^); renal failure (blood urea nitrogen ≥21.4 mmol/L or plasma creatinine level ≥265 μmol/L); pulmonary oedema (oxygen saturation <90% and bibasal crepitations); generalized convulsions; acidosis (venous bicarbonate <15 mmol/L); hyperparasitaemia (peripheral parasitaemia >10%); hyperlactataemia (venous lactate >4 mmol/L); jaundice (bilirubin >43 μmol/L and a parasite count > l00,000/mm^3^); hypoglycaemia (glucose <2.2 mmol/L); and spontaneous bleeding or shock (systolic blood pressure <80 mmHg with cool extremities). Patients were excluded if they were <14 years of age or if they had received parenteral anti-malarial treatment for >48 hours before enrolment. Disease severity was defined by the number of these criteria that a patient satisfied. The independent prognostic utility of the admission platelet count was compared with the widely validated RCAM score [20-22] calculated from the patients’ respiratory rate and GCS on admission (Table 1). Major bleeding was defined as bleeding which resulted in death or necessitated blood transfusion.

**Table 1:** **Calculation of the RCAM score**

	**Score**		
**Variable**	**0 (Normal)**	**1 (Abnormal)**	**2 (Very abnormal)**
Glasgow coma scale	15	11 to 14	≤10
Respiratory rate	<20	20 to 39	≥40

RCAM (0 to 4) is calculated as the respiratory rate score (0 to 2) plus the GCS score (0 to 2).

On enrolment, a history was taken, a physical examination performed and venous blood collected. In Vietnam, patients were randomized to receive intramuscular quinine (n = 271) or intramuscular artemether (n = 279); all the Bangladeshi and Indian patients received intravenous artesunate. Patients received supportive care as per contemporary WHO treatment guidelines [11,23,24]. Access to mechanical ventilation was limited in Vietnam; access to both mechanical ventilation and renal replacement therapy was limited in Bangladesh. Platelet transfusion was not available at any of the sites. The peripheral parasite count (parasites/μL) on admission was calculated from the thin film using the formula: parasite count/1,000 red blood cells × 125.6 × haematocrit (%); or from the thick film using the formula: parasite count/200 white blood cells × 40. In Vietnam haematological indices were manually determined in the hospital laboratory; in Bangladesh and India they were measured with an automated analyser in local private laboratories.

In Vietnam the hospital laboratory performed most biochemical analysis, but plasma glucose and lactate were measured on the ward using dedicated online analysers (Analox, Middlesborough, United Kingdom). Biochemical indices were measured in Bangladesh and India using portable handheld analysers (i-Stat, Abbott, Princeton, New Jersey, USA). Plasma was processed and stored at −80°C for analysis of other laboratory parameters: plasma *Plasmodium falciparum* histidine rich protein 2 (*Pf*HRP2), a measure of parasite biomass, was measured in Bangkok, Thailand, using ELISA (Cellabs, Sydney, New South Wales, Australia), according to the manufacturer’s instructions with minor modifications [25]; angiopoietin-2 (Ang-2), a measure of endothelial activation, was measured in Darwin, Australia, using ELISA (R&D Systems, Minneapolis, Minnesota, USA) [26].

In the Bangladeshi and Indian patients video recordings of blood flow in the microcirculation were collected with an orthogonal polarizing spectral (OPS) imaging device (either Cytoscan from Cytometrics, Heathpark Honiton, Devon, United Kingdom or Microscan from Microvision Medical, Amsterdam, the Netherlands) and quantified using image analysis software (Open Lab 3.1.5, Improvision, Waltham, Massachusetts, USA) as described previously [17].

### Statistics

Data were analysed using statistical software (Stata version 10, StataCorp, College Station, Texas, USA). Correlation coefficients were determined using Spearman’s method. Groups were analysed using the Kruskal-Wallis test and the chi-squared test. Logistic regression was performed where necessary and to control for any influence of the study site or the administered anti-malarial therapy.

### Ethics review

All of the studies received prospective ethical approval from OXTREC (Oxford Tropical Research Ethics Committee) and local ethics review bodies. The ethics and scientific-review committee of The Center for Tropical Diseases in Ho Chi Minh City approved the Vietnamese study, the Bangladeshi Medical Research Council approved the Bangladeshi studies and the institutional ethical board of Ispat General Hospital approved the Indian studies.

## Results

### Patients

Of the 560 Vietnamese patients, 538 (96.1%) had a platelet count documented on admission. The 22 patients who did not have a platelet count recorded had neither more severe disease (*P* = 0.4), nor were more likely to die (*P* = 0.12). Of the 142 Bangladeshi and Indian patients, 109 (76.8%) had an admission platelet count recorded; 31 of the 33 patients with a missing value were studied in 2004 and 2005 when platelet counts were not routinely collected for the studies. The two remaining Bangladeshi and Indian patients without a documented platelet count (from 2011 and 2008 respectively) both survived.

The baseline characteristics of the 647 patients with a platelet count recorded on admission are presented in Table 2. Overall, 107 patients (16.5%) died before discharge; the case-fatality rate was higher in the Bangladeshi and Indian patients than the Vietnamese patients (30 out of 109 (27.5%) versus 77 out of 538 (14.3%); odds ratio (OR) (95% confidence interval (CI)): 2.27 (1.40 to 3.69), *P* = 0.001), although the difference was not significant when controlled for disease severity (OR (95% CI): 1.46 (0.84 to 2.52), *P* = 0.18).

**Table 2:** **Baseline characteristics of the patients**

**Variable**	**n**	**All (n = 647)**	**Survived (n = 540)**	**Died (n = 107)**	***P***
Age (years)	646	30 (22 to 42)	30 (22 to 40)	34 (26 to 45)	0.003
Sex (% male)	646	483 (75%)	409 (76%)	74 (69%)	0.14
Temperature (°C)	647	38.3 (37.5 to 39.0)	38.3 (37.5 to 39.0)	38 (37 to 39.0)	0.04
Mean arterial pressure (mmHg)	646	76.7 (70 to 86.7)	76.7 (70 to 86.7)	79.2 (66.9 to 90.0)	0.31
Heart rate (beats/minute)	641	106 (96 to 120)	105 (95 to 120)	113 (100 to 123)	0.01
Respiratory rate (breaths/minute)	642	28 (24 to 32)	28 (24 to 32)	32 (28 to 40)	<0.001
Glasgow coma scale score	647	10 (8 to 15)	10 (8 to 15)	9 (7 to 13)	<0.001
Haematocrit (%)	646	30 (24 to 36)	30 (24 to 36)	30 (23 to 36)	0.95
Reticulocyte count (%)	514	0.8 (0.4 to 1.2)	0.8 (0.4 to 1.2)	0.5 (0.3 to 1.1)	0.008
Reticulocyte index	517	0.5 (0.3 to 0.8)	0.5 (0.3 to 0.9)	0.4 (0.2 to 0.7)	0.01
White cell count (× 10^9^/L)	643	8.7 (6.2 to 12)	8.1 (6 to 11.2)	11.8 (9.1 to 16.3)	<0.001
Platelet count (× 10^9^/L)	647	48 (30 to 72)	50 (34 to 78)	30 (22 to 52)	<0.001
Mean platelet volume (fL)	34	11.4 (10.4 to 12.4)	11.5 (10.4 to 12.4)	11.3 (11.2 to 12.6)	0.82
Parasite count (parasites/mL)	639	90.4 (18.5 to 327.6)	78.6 (16.1 to 326.7)	101.8 (33.3 to 373.3)	0.09
Sequestration (% blocked capillaries)	107	13.3 (3.3 to 31.5)	10 (2.9 to 26.6)	23.2 (10.0 to 44.0)	0.008
*Pf*HRP2 (ng/mL)	109	2,351 (1,198 to 4,805)	2,187 (976 to 4,216)	3,275 (1,479 to 6,406)	0.07
Lactate (mmol/L)	640	3.5 (2.0 to 4.6)	3.2 (2.0 to 4.6)	5.7 (3.4 to 9.0)	<0.001
Base deficit (mEq/L)	395	5 (2 to 10)	5 (2 to 8)	11 (7 to 17)	<0.001
Anuric on admission	565	51/565 (9%)	35/483 (7.3%)	16/82 (19.5%)	<0.001
Shock on admission	647	47/647 (7.3%)	27/540 (5%)	20/107 (18.7%)	<0.001
Pulmonary oedema on admission	646	15/646 (2.3%)	8/539 (1.5%)	7/107 (6.5%)	0.002
Bleeding on admission	646	34/646 (5.2%)	26/539 (4.8%)	8/107 (7.5%)	0.26
Sodium (mmol/L)	460	133 (127 to 138)	133 (127 to 138)	135 (130 to 140)	0.02
Potassium (mmol/L)	525	4.0 (3.6 to 4.5)	4.0 (3.5 to 4.5)	4.2 (3.7 to 4.8)	0.02
Creatinine (μmol/L)	644	176 (123 to 282)	176 (123 to 254)	239 (146 to 441)	<0.001
Total bilirubin (μmol/L)	544	57.8 (30.6 to 146.2)	51 (27.2 to 119.0)	146.2 (52.7 to 248.2)	<0.001
Direct bilirubin (μmol/L)	420	27.2 (13.6 to 71.4)	23.8 (13.6 to 64.6)	62.9 (20.4 to 127.6)	<0.001
Indirect bilirubin (μmol/L)	420	34.8 (17.0 to 74.8)	34 (14.4 to 64.6)	73.1 (40.4 to 102.3)	<0.001
Angiopoietin-2 (ng/mL)	152	18.2 (10.2 to 29.8)	13.5 (9.4 to 25.1)	25 (14.8 to 41.0)	<0.001

The values represent the median (interquartile range) and absolute number (%). *Pf*HRP2: *Plasmodium falciparum* histidine rich protein 2.

**Figure 1:**
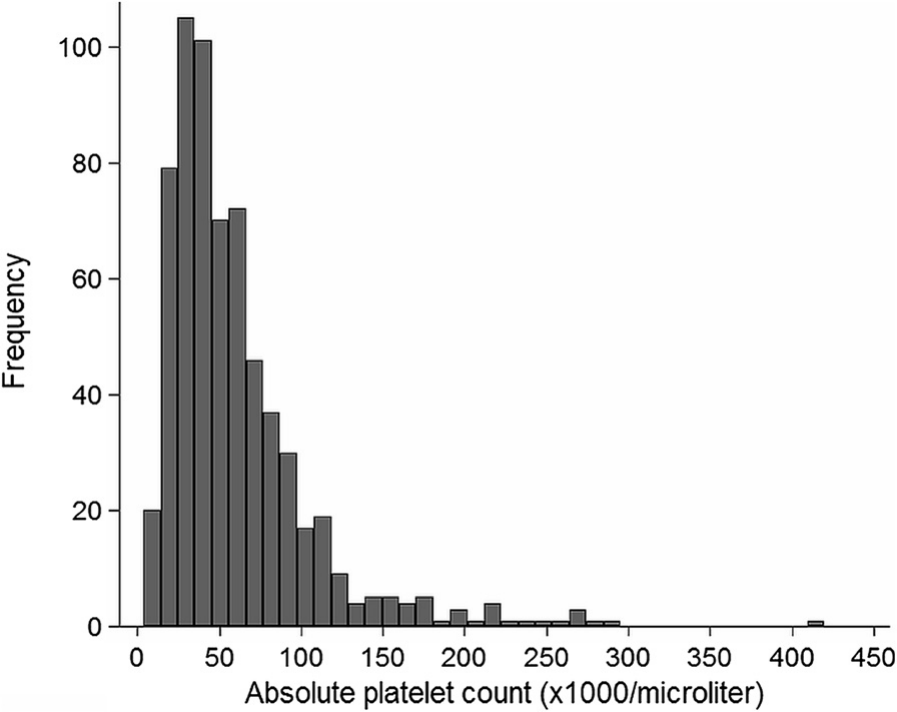
**The peripheral platelet count of the patients on admission to hospital.**

Most patients (614 out of 647, 94.9%) were thrombocytopenic (platelet count <150 × 10^9^/L) on admission; 328 (50.7%) had a platelet count <50 × 10^9^/L (Figure 1). The mean platelet volume was only available in 34 patients but was generally increased: median (IQR) 11.4 (10.4 to 12.4) fL (normal range 7.2 to 11.7 fL).

### Association with bleeding complications

Bleeding was present on admission in 20 out of 328 patients (6.1%) with a platelet count of <50 × 10^9^/L and 14 out of 319 patients (4.4%) with a count ≥50 × 10^9^/L (*P* = 0.33). Bleeding developed in a further 60 out of 328 patients (18.3%) with an admission platelet count of <50 × 10^9^/L and 37 out of 319 patients (11.6%) with a count ≥50 × 10^9^/L (*P* = 0.02). Major bleeding was not common, occurring at some point in the admission of 50 (7.7%) of the patients (30 deaths, 20 additional patients requiring blood transfusion). Although the admission platelet count was lower in patients with major bleeding (median (IQR, range): 40 (28 to 60, 9 to 420) × 10^9^/L) than in patients without major bleeding (50 (30 to 75, 4 to 290) × 10^9^/L; *P* = 0.02), it failed to identify either population reliably (Table 3). Patients dying with bleeding complications rarely died from bleeding alone; these patients also usually had multi-organ dysfunction and satisfied a median (IQR) of 3 (2 to 4) other WHO severity criteria on admission. Similarly the patients with bleeding that required blood transfusion were also frequently anaemic on enrolment (median (IQR) haematocrit 26% (23 to 32%)); many would have required blood transfusion even if bleeding had not supervened. Major bleeding episodes were most commonly gastrointestinal in origin. Proton pump inhibitor therapy was not available at the time of the Vietnam study and acid suppression therapy was administered rarely prior to hospitalisation at any of the study sites.

**Table 3:** **Association between the admission platelet count, bleeding complications and outcome**

**Platelet count on admission (× 10** ^**9**^ **/L)**	**n**	**Any bleeding during hospitalisation**	**Died with bleeding complications**	**Survived but required blood transfusion for bleeding**	**Major bleeding** ^**a**^	**Died from any cause**
Less than 20	39	6 (15%)	0	3 (7.7%)	3 (7.7%)	18 (46%)
20 to 49	289	74 (26%)	20 (6.9%)	9 (3.1%)	29 (10 %)	59 (20%)
50 to 99	235	46 (20%)	9 (3.8%)	6 (2.6%)	15 (6.4%)	24 (10%)
≥100	84	5 (6%)	1 (1.2%)	2 (2.4%)	3 (3.6%)	6 (7.1%)
Total	647	131(20%)	30 (4.6%)	20 (3.1%)	50 (7.7%)	107 (17%)

^a^ Patients who died from bleeding complications or who had bleeding and received blood transfusion.

### Association with disease severity, parasite burden and measures of endothelial function

The admission platelet count correlated inversely with disease severity (r_s_ = −0.21, *P* <0.001) and the circulating parasite count (r_s_ = −0.29, *P* <0.001). Measures of sequestration (OPS imaging data) and total parasite biomass (*Pf*HRP2) were only available in Bangladeshi and Indian patients; however, both correlated inversely with the admission peripheral platelet count (r_s_ = −0.31, *P* = 0.001 and r_s_ = −0.28, *P* = 0.003, respectively). Admission plasma Ang-2 concentrations were available in 152 cases (92 Bangladeshi and 60 Vietnamese patients) and were markedly elevated (median (IQR) 18.2 (10.3 to 29.8) ng/mL). However, an inverse correlation between plasma Ang-2 concentrations and the peripheral platelet count failed to reach statistical significance (r_s_ = −0.15, *P* = 0.059) (Table 4).

**Table 4:** **Association between the platelet count and clinical and other laboratory indices on admission**

**Variable**	**Platelets ≥ 50 × 10** ^**9**^ **/L n = 319**	**Platelets <50 × 10** ^**9**^ **/L n = 328**	***P***
Died	30/319 (9.4%)	77/328 (23.5%)	<0.001
Age (years)	28 (22 to 38)	33 (24 to 45)	<0.001
Sex (% male)	237/319 (74.0%)	246/327 (75.2%)	0.79
Temperature (°C)	38.5 (37.5 to 39.0)	38.1 (37.5 to 39.2)	0.74
Mean arterial pressure (mmHg)	76.7 (70.0 to 86.7)	78.3 (70.0 to 90.0)	0.05
Heart rate (beats/minute)	100 (96 to 120)	110 (95 to 120)	0.09
Respiratory rate (breaths/minute)	28 (24 to 32)	28 (24 to 36)	0.006
Glasgow Coma Scale Score	10 (8 to 15)	11 (8 to 15)	0.11
Haematocrit (%)	28 (23 to 35)	31 (25 to 38)	<0.001
White cell count (× 10^9^/L)	8.8 (6.3 to 11.6)	8.6 (6.1 to 12.6)	0.75
Reticulocyte count (%)	0.9 (0.5 to 1.5)	0.6 (0.3 to 1.0)	<0.001
Parasite count (parasites/mL)	42.2 (10.4 to 184.3)	151.6 (32.3 to 479.9)	<0.001
Sequestration (% blocked capillaries)	6.6 (1.7 to 18.3)	16.6 (6.6 to 34.9)	0.01
*Pf*HRP2 (ng/mL)	1,739 (783 to 3,203)	2,834 (1,482 to 5,209)	0.01
Lactate (mmol/L)	2.9 (1.9 to 4.4)	4.0 (2.4 to 6.3)	<0.001
Base deficit (mEq/L)	4 (1 to 8)	6 (3 to 11)	<0.001
Creatinine (μmol/L)	164 (115 to 264)	185 (138 to 310)	0.007
Total bilirubin (μmol/L)	44.2 (23.8 to 115.6)	74.8 (40.8 to 170.0)	<0.001
Indirect bilirubin (μmol/L)	26.5 (13.6 to 54.4)	47.6 (23.8 to 85.0)	<0.001
Angiopoietin-2 (ng/mL)	16.3 (9.4 to 30.2)	19.4 (11.6 to 29.8)	0.14
Anuric on admission	20/289 (6.9%)	31/276 (11.2%)	0.07
Shock on admission	19/319 (6.0%)	28/328 (8.5%)	0.21
Pulmonary oedema on admission	6/319 (1.9%)	9/327 (2.8%)	0.46

The values represented the median (interquartile range) and absolute number (%). *Pf*HRP2: *Plasmodium falciparum* histidine rich protein 2.

### Association with outcome and independent prognostic utility

**Figure 2:**
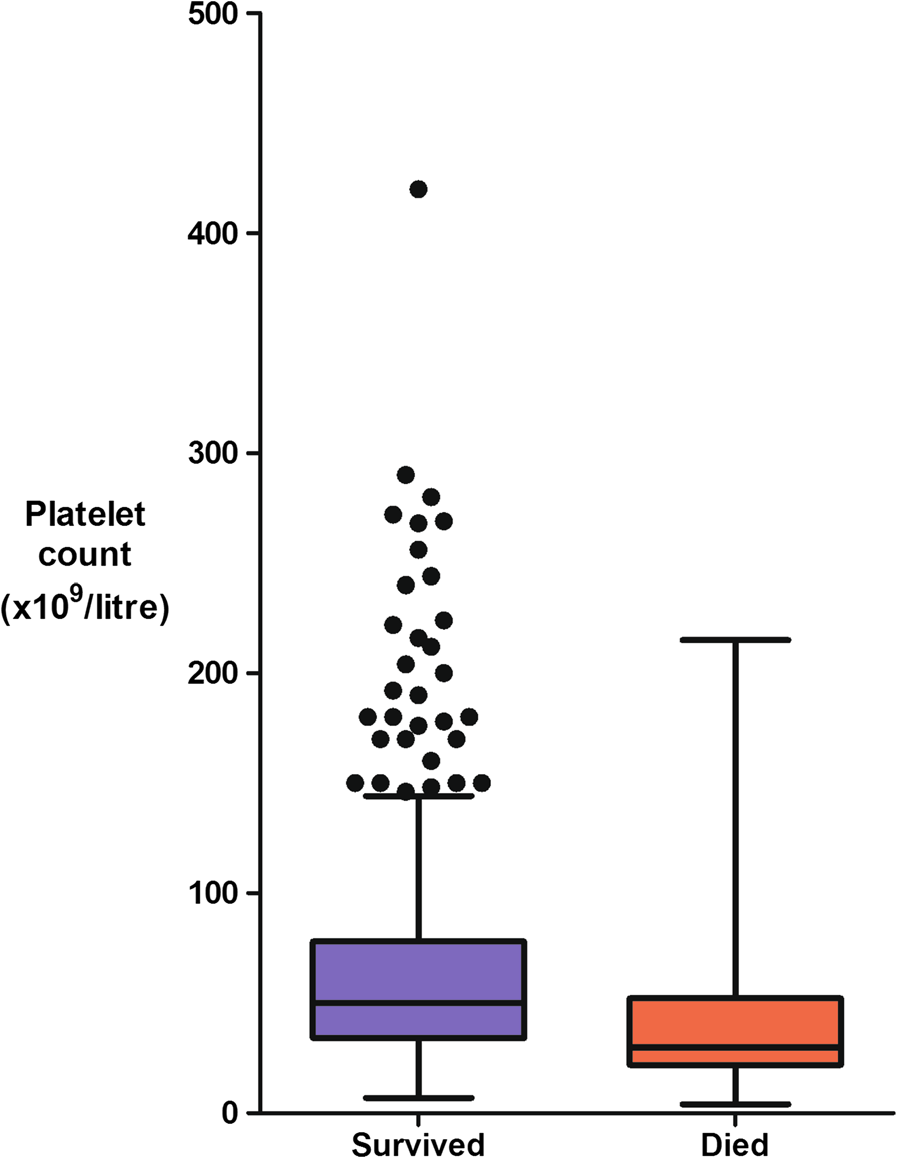
**The relationship between the platelet count on admission and outcome.**

The admission platelet count correlated with the risk of death in both datasets. Overall, the median (IQR) platelet count in the patients who died was 30 (22 to 52) × 10^9^/L compared to 50 (34 to 78) × 10^9^/L in survivors (*P* = 0.0001) (Figure 2). The case-fatality rate increased with declining platelet count (Table 3): 77 out of 328 patients (23%) with severe thrombocytopenia (<50 × 10^9^/L) died (OR (95% CI): 2.96 (1.88 to 4.66), although when considered with other laboratory indices of disease severity, severe thrombocytopenia was not independently predictive of outcome (Table 5). Of the 39 patients with profound thrombocytopenia (<20 × 10^9^/L), 18 (46%) died (OR: 5.00 (95% CI 2.56 to 9.75). However, these patients were usually relatively easy to identify: the median (IQR) GCS of these 39 patients was 8 (6 to 12) and the median (IQR) respiratory rate was 33 (26 to 44) breaths per minute. The 18 patients with profound thrombocytopenia on admission who later died concurrently satisfied a median (IQR) of 3 (3 to 4) WHO severity criteria and had pronounced derangement of other laboratory indices (median (IQR) plasma lactate, 6 (3.6 to 9.3) mmol/L; base deficit, 12 (9 to 17) mEq/L; plasma creatinine, 229 (140 to 453) μmol/L; and total bilirubin, 195 (105 to 260) μmol/L).

**Table 5:** **Independent ability of laboratory variables measured on admission to predict outcome**

**Variable**	**Cut-off**	**Number** ^**a**^	**Odds ratio**	**95% Confidence interval**	***P***
White cell count	>11 × 10^9^/L	195 (30%)	2.74	1.53 to 4.89	0.001
Plasma creatinine	>265 μmol/L	176 (27%)	2.40	1.3 to 4.43	0.005
Plasma lactate	>4 mmol/L	260 (41%)	2.25	1.26 to 4.02	0.006
Total bilirubin	>51 μmol/L	313 (57%)	2.26	1.13 to 4.50	0.02
Platelet count	<50 × 10^9^/L	328 (51%)	1.75	0.96 to 3.18	0.07
Reticulocyte count	<0.5%	190 (37%)	1.19	0.63 to 2.26	0.59

^a^Number (%) of patients with a value meeting the cut-off. The denominator for the calculation of the percentage was the number of patients in the dataset with a measurement available (not all patients had data for every variable). Multivariable logistic regression with death as the dependent variable; explanatory variables chosen on the basis of a significant association with outcome in univariate analysis (with *P* <0.01 allowing for multiple comparisons, see Table 2). The cut-offs were chosen based on the WHO severity criteria cut-off (for those that are WHO severity criteria: creatinine, lactate and bilirubin). For those that are not WHO severity criteria, the cut-offs were based on the reference range (white blood cell count and reticulocyte count) and previous studies (platelet count). Base deficit was not included in the model as there were fewer data for this variable; acidosis was represented by plasma lactate.

**Figure 3:**
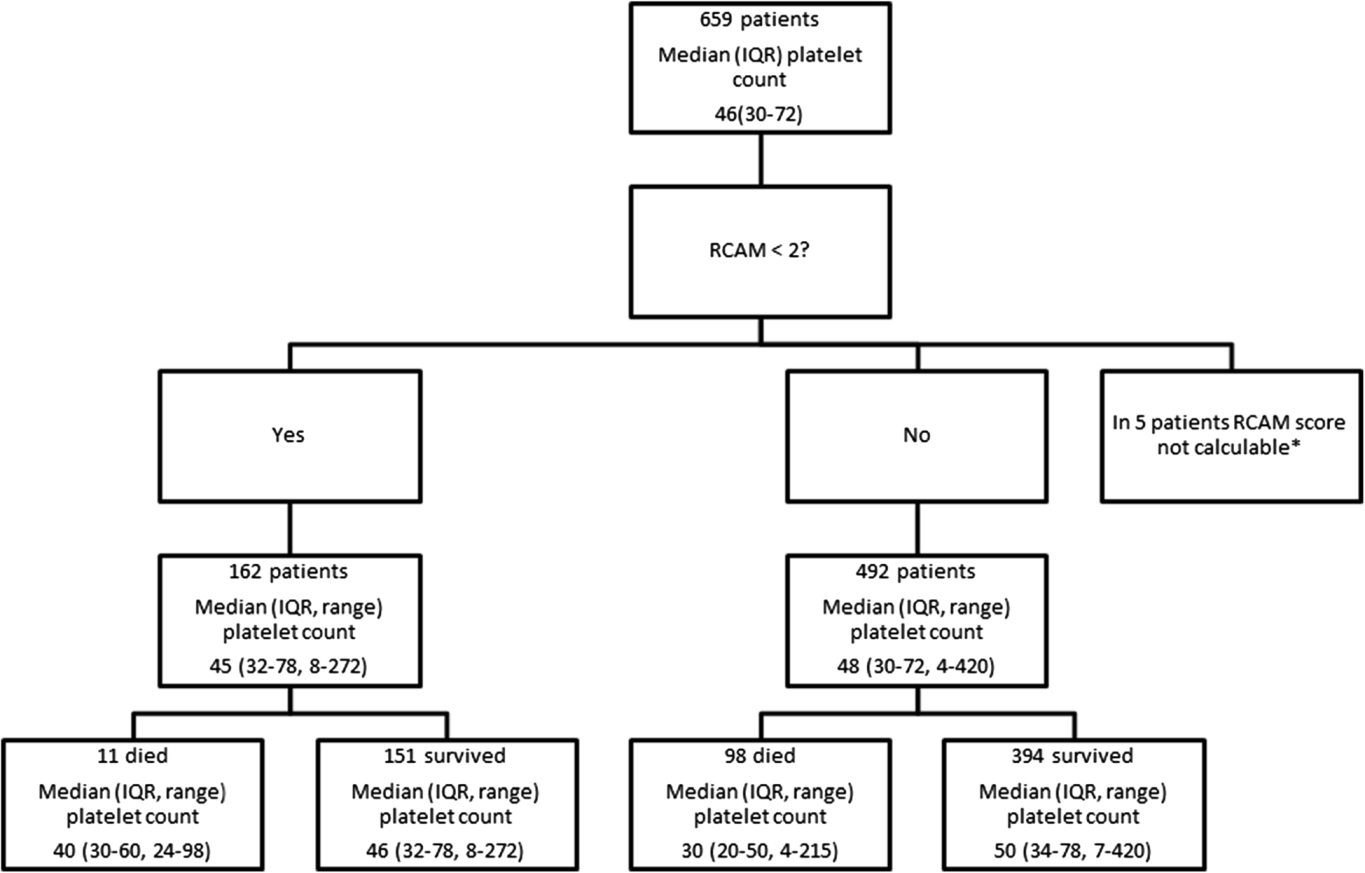
Ability of the platelet count on admission to predict outcome when compared the simple RCAM score. *In five patients the respiratory rate on admission was not recorded. IQR, interquartile range.

In total, 642 patients (99.2%) had data allowing retrospective calculation of a RCAM score on admission; 104 of these patients died, 93 (89.4%) of whom had a RCAM score ≥2. The platelet count did not improve the identification of the 11 patients who died but were classified as low-risk by the RCAM score (RCAM score <2). The platelet counts of these 11 patients (median (IQR, range): 40 (30 to 60, 24 to 98) × 10^9^/L)) were not statistically or clinically significantly different to the 151 patients with an RCAM score <2 who survived: 48 (32 to 78, 8 to 272) × 10^9^/L) (*P* = 0.56) (Figure 3). Of the patients with profound thrombocytopenia (<20 × 10^9^/L), 34 out of 39 (87.2%) had a RCAM score ≥2; the five patients with profound thrombocytopenia but a RCAM score <2 all survived.

## Discussion

In this multi-centre study of adults with severe falciparum malaria, the platelet count on admission correlated with disease severity and outcome, but had limited independent prognostic utility when considered with other laboratory indices. Knowledge of the platelet count offered no significant prognostic advantage over determination of the respiratory rate and GCS. The platelet count on admission was lower in patients who went on to develop major bleeding, but was unable to identify this population reliably.

Thrombocytopenia was more frequent in this series than in previous studies of adults with falciparum malaria [2,3,27], presumably as a result of a strict case definition leading to the enrolment of patients with more severe disease. The increased mean platelet volume, although available in few patients, suggests the presence of young, functional platelets, and likely explains the relatively low incidence of major bleeding - despite the profound thrombocytopenia - seen in the study. This low incidence of major bleeding and the significant challenges associated with the delivery of blood products in the resource-poor setting [28] argues strongly against the routine use of platelet transfusions in these patients. The prompt and spontaneous recovery seen in the platelet counts of this patient population provides additional support for this clinical approach [29].

After an association between thrombocytopenia and outcome was demonstrated in a large Indonesian population-based study of malaria, it was suggested that a platelet count of less than 20 × 10^9^/L should be used as a severity criterion in malaria and that the platelet count might have use in patient triage [12]. An Indian hospital-based study reached similar conclusions [13]. A platelet count has the virtue of being easier to measure than some other recognized laboratory predictors of mortality, such as plasma lactate and base deficit. Our series confirms that profound thrombocytopenia should alert clinicians managing patients with malaria - patients with a platelet count below 20 × 10^9^/L were five times more likely to die than patients with a higher platelet count. However, if clinicians in this series had used the platelet count to triage patients, it would not have resulted in significant changes in their management. None of the patients who would later die despite being classified as low-risk with the simple bedside RCAM score had an admission platelet count below 20 × 10^9^/L. Thus, whilst there is an association between the presence of profound thrombocytopenia and the likelihood of a complicated course, the addition of another severity criterion to the already complex definition of severe falciparum malaria [30] may not assist clinicians substantially.

OPS imaging has been used to demonstrate microvascular obstruction *in vivo* in adults with falciparum malaria [15]. The correlation seen in this series between microvascular obstruction and the circulating platelet count may represent the diffuse sequestration of radiolabelled platelets seen in thrombocytopenic patients with falciparum malaria [5]. Certainly platelets have been implicated in the pathogenesis of two of the processes (auto-agglutination [31] and cytoadherence [32]) that contribute to microvascular obstruction, and increased numbers of platelets and platelet-fibrin thrombi have been reported in the cerebral microcirculation of paediatric cases of fatal falciparum malaria [8]. However, platelets are notable by their absence from the microcirculation in adult post-mortem series [33,34]. Furthermore thrombocytopenia is also frequent in *Plasmodium vivax* malaria [12,27], a disease in which significant sequestration is absent [35]. Although severe thrombocytopenia is less frequent in vivax malaria, there are many shared findings, including increased mean platelet volumes and a correlation with the circulating parasite count [36], suggesting that there are common pathways in the pathogenesis of the thrombocytopenia in the two infections.

One of these common pathways is likely to be endothelial activation [34,37]. During endothelial activation, activated high-multimeric von Willebrand Factor (vWF) is released from specialized secretory vesicles in endothelial cells known as Weibel-Palade bodies (WPBs) [38]. Once in the circulation, vWF aggregates platelets, resulting in their clearance from the circulation. Studies performed in both falciparum and vivax malaria demonstrate an inverse association between the circulating platelet count and plasma concentrations and activity of vWF [9,10]. Falls in the plasma concentrations of ADAMTS13, a regulating enzyme that catabolizes vWF multimers, are also seen in infections with both species [9,10]. Although vWF and ADAMTS13 were not determined in this series, plasma concentrations of Ang-2 were markedly elevated, suggesting the presence of pronounced systemic endothelial activation. Indeed, Ang-2 is stored in WPBs and is released with vWF during WPB exocytosis.

An interaction between microvascular obstruction and endothelial activation may explain the association between the OPS findings and the circulating platelet count seen in this series [34]. The resting, un-activated endothelium constitutively releases nitric oxide, which helps maintain endothelial quiescence and also directly inhibits platelet aggregation [39,40]. The shear stress of normally flowing blood is the principal signal for this nitric oxide release [40] and it would therefore be expected that microvascular obstruction could lead to endothelial activation via this mechanism. Microvascular obstruction also leads to tissue hypoperfusion and hypoxia - another potent stimulus for endothelial activation. Endothelial activation facilitates further cytoadherence through the upregulation of endothelial receptors that act as ligands for parasitized erythrocytes [34], resulting in further microvascular obstruction and potentially precipitating a vicious cycle.

The study has other noteworthy haematological findings. Erythropoiesis was significantly depressed with a reticulocyte production index of less than 1 in almost 90% of the patients. Accordingly, most patients were anaemic but severe anaemia (haematocrit <20%) was relatively infrequent, a finding that may reflect the fact that this study was performed exclusively in adults [41]. It suggests that prognostic tools that use the platelet count and haemoglobin concentration may have limited utility in the adult population [12]. The median white blood cell count was greater in this cohort than in outpatients with malaria in whom mild leucopenia is typical [42]. The admission white cell count was significantly higher in the patients who died than in survivors, an association that has been recognized previously [13,43].

Our study had significant limitations. Its retrospective design precluded complete data collection. Measurements of vWF and ADAMTS13 concentrations and activity would have defined the extent of this pathway’s contribution to the pathogenesis of thrombocytopenia. Coagulation studies would have defined the contribution of coagulopathy to bleeding complications, although disseminated intravascular coagulation was rarely suspected clinically. The study assessed the prognostic utility of the admission platelet count, but sequential measurement may have improved its ability to predict bleeding complications, in particular. The dataset consisted of patients who had already satisfied a definition of severe malaria; the prognostic utility of the platelet count may be different outside of a study setting. Because the studies only enrolled adults, the findings cannot necessarily be generalized to children.

## Conclusions

Adults with severe falciparum malaria are usually thrombocytopenic, some profoundly so. Despite this, the risk of major bleeding is relatively low. Patients with multi-organ involvement and those who will die before discharge are more likely to have a lower platelet count; however, these patients are more reliably identified by simple clinical assessment and traditional laboratory indices of severe disease.
